# Network pharmacology-based research on the effect of Radix Astragali on osteosarcoma and the underlying mechanism

**DOI:** 10.1038/s41598-023-49597-x

**Published:** 2023-12-15

**Authors:** Yafang Zhang, Junqiang Wei, Lingwei Kong, Mingze Song, Yange Zhang, Xiangyu Xiao, Haiying Cao, Yu Jin

**Affiliations:** Department of Traumatology and Orthopaedics, Affiliated Hospital of Chengde Medical University, Chengde, 067000 Hebei China

**Keywords:** Bone cancer, Sarcoma, Tumour biomarkers, Drug screening, Target identification, Target validation

## Abstract

To explore the anti-tumor effects of Radix Astragali on osteosarcoma and its mechanism. We analyzed the PPI network of Radix Astragali’s potential targets for treating osteosarcoma and got the hub targets. We used KM curves to screen hub targets that could prolong sarcoma patients’ survival time. We performed GO and KEGG enrichment analysis of Radix Astragali’s potential targets and predicted Radix Astragali's molecular mechanism and function in treating osteosarcoma. The binding process between the hub targets, which could prolong sarcoma patients' survival time, and Radix Astragali was simulated through molecular docking. PPI network analysis of potential therapeutic targets discriminated 25 hub targets. The KM curves of the hub targets showed there were 13 hub targets that were effective in improving the 5-year survival rate of sarcoma patients. GO and KEGG enrichment demonstrated that Radix Astragali regulates multiple signaling pathways of osteosarcoma. Molecular docking results indicated that Radix Astragali could bind freely to the hub target, which could prolong the sarcoma patient's survival time. Radix Astragali act on osteosarcoma by regulating a signaling network formed by hub targets connecting multiple signaling pathways. Radix Astragali has the potential to become a drug for treating osteosarcoma and prolonging the sarcoma patient's survival time.

## Introduction

Osteosarcoma is one of the malignant tumors that occur in children and adolescents^1,2^. It originates from primitive mesenchymal stem cells and occurs most often in the epiphysis of long stem bones^3^, such as the distal femur and the proximal tibia^4^. The current treatment strategy for osteosarcoma is neoadjuvant chemotherapy, radical resection, and adjuvant chemotherapy^5^. With the addition of chemotherapy, the overall 5-year survival rate of patients with osteosarcoma has reached approximately 60–70%^6^, but the overall 5-year survival rate decreases to lower than 15%after the occurrence of distant metastasis^7^. Although new drugs for OS treatment, such as immune checkpoint inhibitors and targeted drugs are constantly being explored^8,9^, osteosarcoma’s five-year overall survival rate has not significantly improved in the last few decades^10^. There is still an urgent need to discover new treatment options.

Radix Astragali is one of the traditional Chinese medicines^11^, It is the root of Astragalus memeranaceus (Fisch.) Bge. Var. mongholicus (Bge.) Hsiao, or A. membranaceus (Fisch.) Bge^12^. Existing evidence has proven that Radix Astragali exerts inhibitory effects on tumor cells by inhibiting proliferation, migration, and invasion^11,13,14^. However, research related to the role of Radix Astragali in inhibiting osteosarcoma and its underlying mechanism is insufficient.

Network pharmacology is efficient and cost-effective^15,16^. It explores the relationship between diseases and drugs by integrating systems biology, bioinformatics, and computer science^15,17^. The pharmacology has been transformed from a single-drug-single-target model to a multi-drug-multi-target model^15–18^. Therefore, this experiment systematically investigated the effects and mechanisms of Radix Astragali on osteosarcoma using network pharmacology and molecular docking techniques.

## Methods

**Figure 1:**
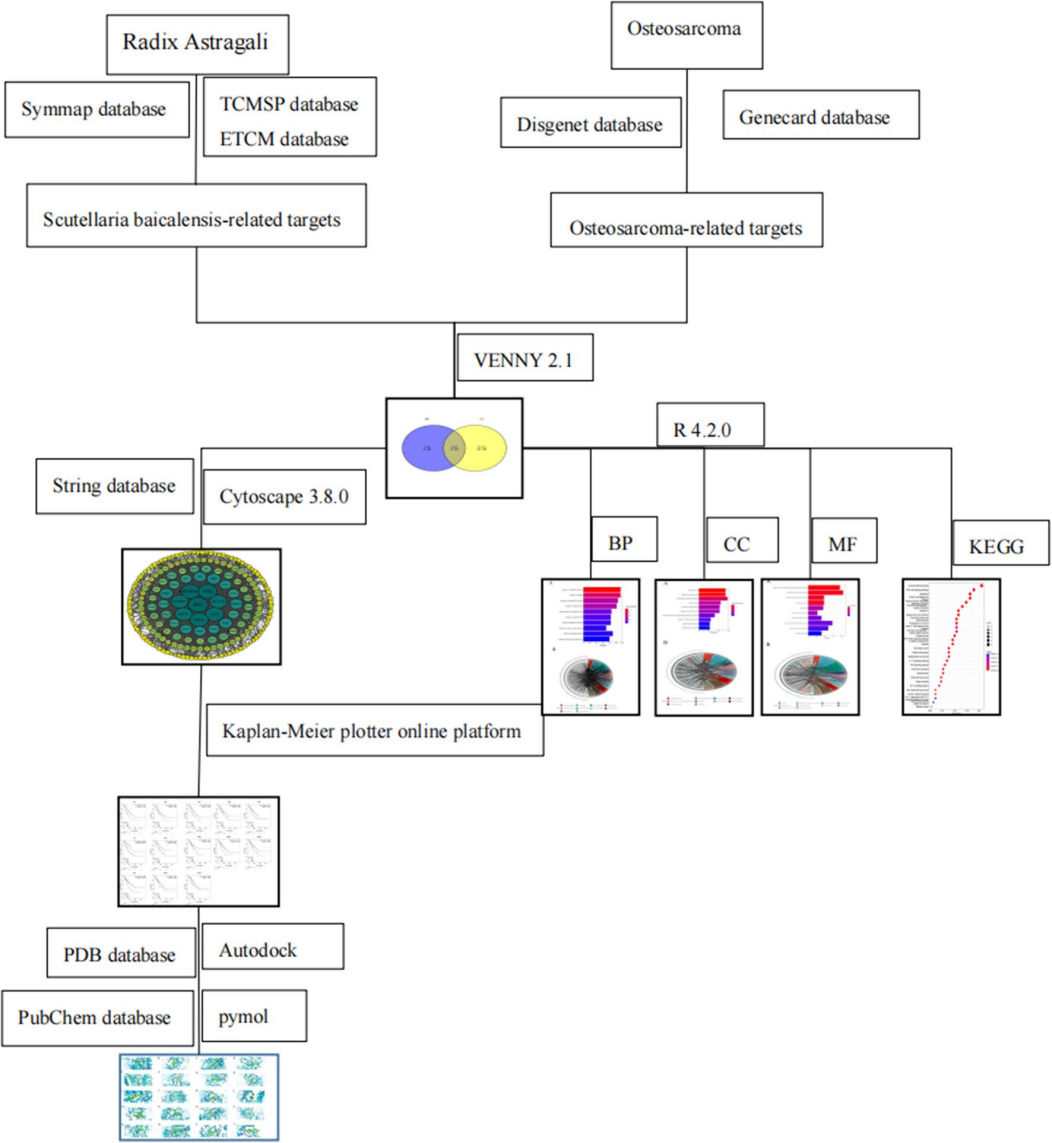
Network pharmacological study of Radix Astragali for the treatment of osteosarcoma schematic diagram.

The flow chart of the study design is shown in Fig. 1.

### Getting the ingredients of Radix Astragali

The drug ingredients of Radix Astragali were obtained from the TCMSP database (https://old.tcmsp-e.com/tcmsp.php) with the keyword "Radix Astragali”. The drug ingredients were screened with the criterias of oral bioavailability (OB) > 30% and drug Likeness (DL) > 0.18. The obtained drug ingredients were regarded as the drug ingredients of Radix Astragali. The TCMSP database (https://old.tcmsp-e.com/tcmsp.php) is a platform on the systemic pharmacology of herbal medicines where we can get the relationship between drugs, targets, and diseases. The database provides information on the identification of drug ingredients, drug target networks, networks of related drug target diseases, and pharmacokinetic properties such as OB and (DL) for natural compounds.

### Collection of Radix Astragali-related targets

Radix Astragali-related targets were screened in the TCMSP database (https://old.tcmsp-e.com/tcmsp.php) based on the drug ingredients of Radix Astragali. Radix Astragali-related targets were screened in the TCMSP database (https://old.tcmsp-e.com/tcmsp.php) based on the drug ingredients of Radix Astragali. Using "Radix Astragali" as the keyword, we searched the ETCM (http://www.tcmip.cn/ETCM/) and Symmap databases (http://www.symmap.org/) for Radix Astragali-related targets. Finally, we integrated and de-duplicated the Radix Astragali-related targets obtained from the above three databases, and the targets obtained were regarded as Radix Astragali-related targets.The ETCM database (http://www.tcmip.cn/ETCM/) is a comprehensive resource database for traditional Chinese medicine that went online in 2018 by the Chinese Academy of Traditional Chinese Medicine, bringing together information on a wide range of herbal medicines, herbal compounding, herbal chemical composition, drug targets, and related diseases. SymMap (Symptom Mapping) (http://www.symmap.org/) is a TCM evidence association database. The database herbs and the corresponding TCM symptoms, and corresponds TCM symptoms to Western medicine symptoms, and includes diseases, herbal ingredients, drug targets associated with these symptoms, as well as the correlations between these six data types.

### Collection of osteosarcoma-related targets

We searched the Genecard database (https://www.genecards.org/) for osteosarcoma-related targets using the keyword "Osteosarcoma", and the targets obtained were osteosarcoma-related targets. Genecards is a comprehensive, searchable database of genes where we can access information on almost all known human genes.

### Construction of protein–protein interaction (PPI) networks

With R software (R 4.2.0), we used Radix Astragali-associated targets and osteosarcoma-associated targets to draw Venn diagrams. The potential targets of Radix Astragali for osteosarcoma were imported into the String database (https://cn.string-db.org/), with species set to "Homo sapiens" and confidence set to "0.9" to construct the PPI network.

### Acquisition of hub targets

We also imported the PPI network into Cytoscape software (3.8.0) to further analyze and obtain the coefficients of the targets, such as degree, betweenness Centrality, and Closeness Centrality, and then screened the targets twice with degree ≥ median degree, betweenness Centrality ≥ median Closeness Centrality, and Closeness Centrality ≥ median Closeness Centrality as the screening criteria. Centrality ≥ Betweenness Centrality and Closeness Centrality ≥ Closeness Centrality were used as the screening criteria to screen the hub targets twice, and the obtained targets were the hub targets of the PPI network.

### Plotting Kaplan–Meier (KM) curves

We imported the hub targets into the Kaplan–Meier Plotter online platform (https://kmplot.com/analysis/) to plot KM curves. Since there is no separate database for osteosarcoma in this platform, we plotted KM curves based on the sarcoma database, and P < 0.05 means statistically significant. The division between high and low groups was chosen as the median for gene expression levels. The Kaplan–Meier Plotter database (https://kmplot.com/analysis/) was constructed based on gene microarray and RNA-seq data from public databases such as GEO, EGA, and TCGA and was used to integrate gene expression information and clinical prognostic values for meta-analysis and the study, discovery, and validation of survival-related molecular markers.

### Gene ontology (GO) and Kyoto encyclopedia of genes and genomes (KEGG) enrichment analysis

To analyze the potential functions and pathways of potential targets of Radix Astragali for osteosarcoma treatment acting on osteosarcoma, we used R software (R 4.2.0) to perform GO and KEGG enrichment analysis of potential targets of Radix Astragali for osteosarcoma treatment, and to illustrate GO enrichment in terms of biological processes, cellular components, and molecular functions. R-package-Bioconductor Cluster Profiler is an R package (R × 64 4.0.3) widely used for gene bioinformatics analysis.

### Molecular docking

Autodock software is one of the software used for molecular docking. In this experiment, two sub-software, Autodock 4 (4.2.6) and Autodock Vina (1.1.2), were used for molecular docking, and Pymol software (2.5) was used for molecular pre-processing and visualization of docking results. The hub targets whose differential expression impacts the survival of sarcoma patients and the corresponding drug ingredients were used to perform molecular docking. 3D structures of the targets were obtained from the The Protein Data Bank (PDB) database (https://www.rcsb.org/) and 3D structures of the drug ingredients from the PubChem database (https://pubchem.ncbi.nlm.nih.gov/), and the target proteins and drug ingredients were pre-processed using Autodock 4 (4.2.6) and Pymol software (2.5), and Autodock Vina software (1.1.2) was used as batch molecular docking.

### Ethical approval

Because we use public and anonymous data, according to the ethics guidelines, neither informed consent nor approval of the ethics committee is required.

## Results

### Radix Astragali-related targets

The drug ingredients of Radix Astragali were searched in the TCMSP database (https://old.tcmsp-e.com/tcmsp.php) using the keyword "Radix Astragali," and the drug ingredients were screened using the screening criteria of OB > 30% and DL > 0.18. We finally obtained 20 drug ingredients of Radix Astragali (Table 1).

**Table 1:** Characteristics of the active ingredients.

Mol ID^1^	Molecule name	OB^2^ (%)	DL^3^	MW^4^
MOL000211	Mairin	55.38	0.78	456.78
MOL000239	Jaranol	50.83	0.29	314.31
MOL000296	Hederagenin	36.91	0.75	414.79
MOL000033	(24S)-24-Propylcholesta-5-Ene-3beta-Ol	36.23	0.78	428.82
MOL000354	Isorhamnetin	49.6	0.31	316.28
MOL000371	3,9-di-O-methylnissolin	53.74	0.48	314.36
MOL000374	5′-hydroxyiso-muronulatol-2′,5′-di-O-glucoside	41.72	0.69	642.67
MOL000378	7-O-methylisomucronulatol	74.69	0.3	316.38
MOL000379	9,10-dimethoxypterocarpan-3-O-D-glucoside	36.74	0.92	462.49
MOL000380	(6aR,11aR)-9,10-dimethoxy-6a,11a-dihydro-6H-benzofurano[3,2-c]chromen-3-ol	64.26	0.42	300.33
MOL000387	Bifendate	31.1	0.67	418.38
MOL000392	Formononetin	69.67	0.21	268.28
MOL000398	Isoflavanone	109.99	0.3	316.33
MOL000417	Calycosin	47.75	0.24	284.28
MOL000422	Kaempferol	41.88	0.24	286.25
MOL000433	FA	68.96	0.71	441.45
MOL000438	(3R)-3-(2-hydroxy-3,4-dimethoxyphenyl)chroman-7-ol	67.67	0.26	302.35
MOL000439	Isomucronulatol-7,2′-di-O-glucosiole	49.28	0.62	626.67
MOL000442	1,7-Dihydroxy-3,9-dimethoxy pterocarpene	39.05	0.48	314.31
MOL000098	Quercetin	46.43	0.28	302.25

^1^*ID* identity, ^2^*OB* oral bioavailability, ^3^*DL* drug likeness, ^4^*MW* molecule weight.

### Radix Astragali-related targets

209 Radix Astragali-related targets were obtained from the TCMSP database (https://old.tcmsp-e.com/tcmsp.php), 272 Astragalus-related targets were obtained from the ETCM database (http://www.tcmip.cn/ETCM/) and 100 Radix Astragali-related targets were obtained from the Symmap database (http://www.symmap.org/). Each database has a different focus, and there are differences between databases, therefore, there may be potential risks in the joint analysis of multiple databases, We compensate for the absence of some targets in a single database by merging targets from multiple databases. The above targets were integrated and de-weighted to obtain 451 Radix Astragali-related targets as the final Radix Astragali-related targets for further processing.

### Osteosarcoma-related targets

We searched the Genecard database (https://www.genecards.org/) for osteosarcoma-related targets using the keyword "osteosarcoma" and obtained 5347 targets, which were considered osteosarcoma-related targets for subsequent data processing.

### PPI network

**Figure 2:**
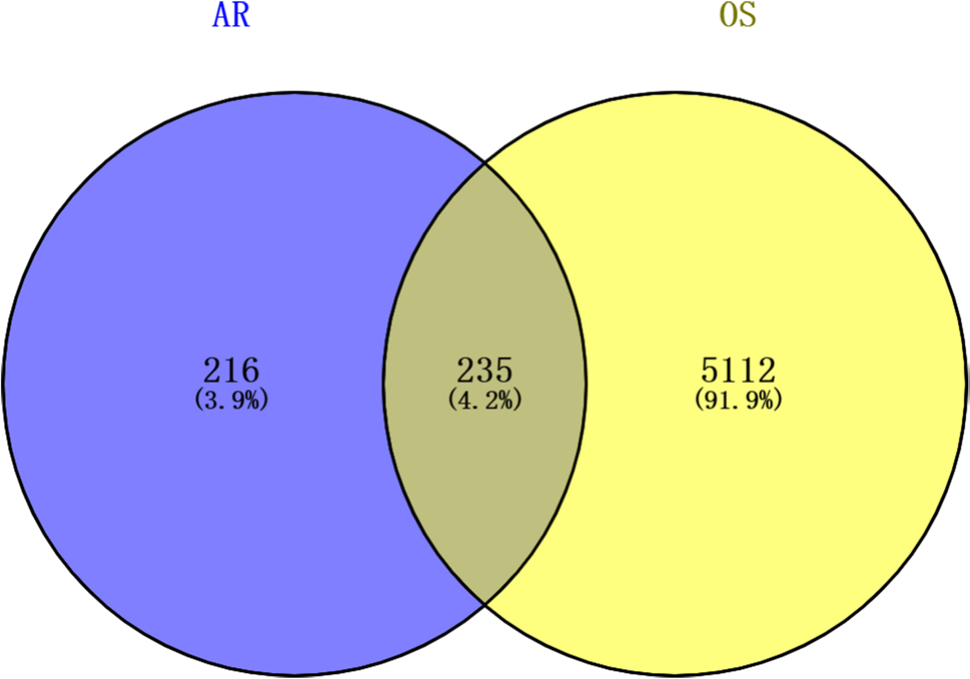
The venn diagram about the target of Radix Astragali and the target of osteosarcoma. The blue circle represents the target of Radix Astragali, and the yellow circle represents the target of osteosarcoma, the intersection of the two circles represents the target of Radix Astragali for osteosarcoma.

**Figure 3:**
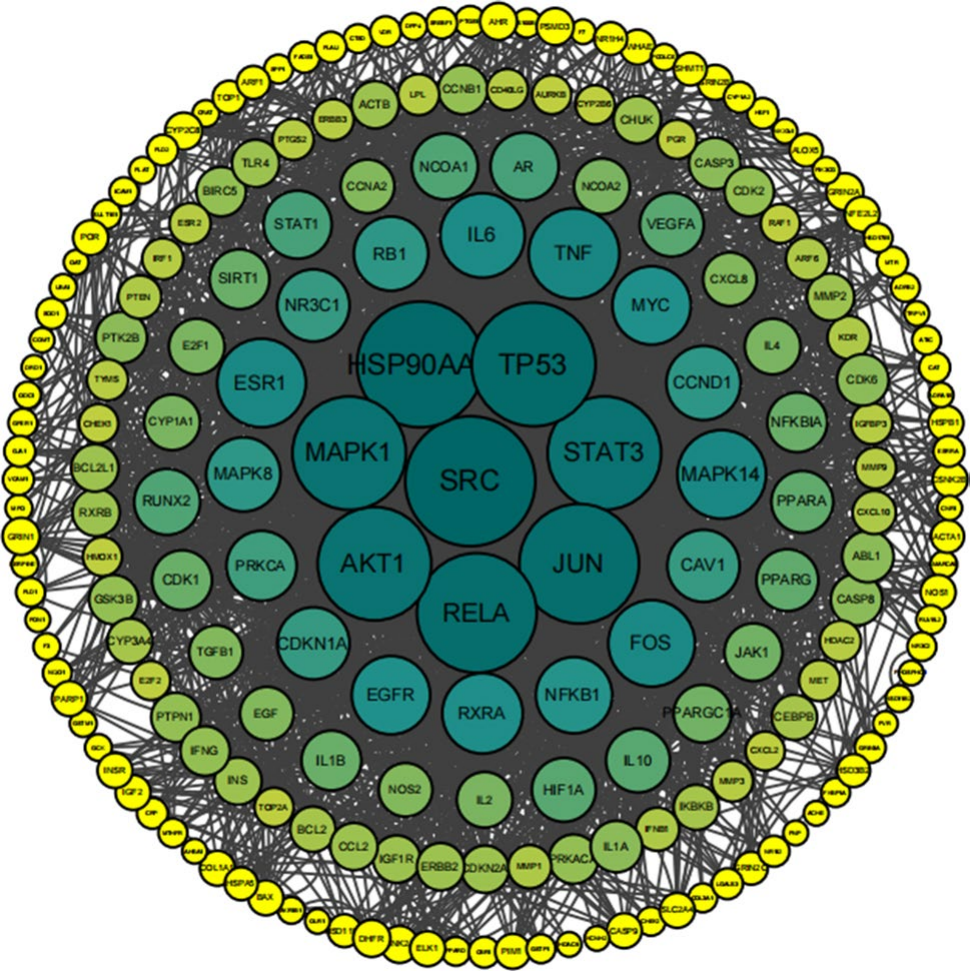
PPI network of Radix Astragali in the treatment of osteosarcoma. The nodes represent potential therapeutic targets of Radix Astragali against osteosarcoma. The larger the node and darker the color, the higher the corresponding target degree and the more connections to other nodes.

We constructed Venn diagrams with Radix Astragali-related targets and osteosarcoma-related targets (Fig. 2). The targets in the intersection part of the two were potential targets of Radix Astragali for osteosarcoma, and a total of 235 potential targets of Radix Astragali for osteosarcoma were obtained. The potential targets of Radix Astragali for osteosarcoma were imported into the String database (https://cn.string-db.org/) to construct a PPI network and imported into Cytoscape software (3.8.0) for processing and analysis (Fig. 3), with degree ≥ 36, Betweenness Centrality ≥ 0.024538651 and Closeness Centrality ≥ 0.431623932 as the screening criteria for screening pivotal targets were obtained, and a total of 25 hub targets were obtained (Table 2).

**Table 2:** Characteristics of the hub gene.

Name	Betweenness centrality	Closeness centrality	Degree
SRC	0.155514042	0.5	104
HSP90AA1	0.092403433	0.48441247	100
TP53	0.099826527	0.495098039	98
JUN	0.054483579	0.492682927	94
RELA	0.05164348	0.489104116	94
AKT1	0.075451214	0.492682927	88
MAPK1	0.047748112	0.482100239	88
STAT3	0.049144241	0.477541371	88
TNF	0.026999842	0.433476395	66
ESR1	0.026659048	0.464367816	64
MAPK14	0.017013089	0.458049887	64
FOS	0.025847927	0.463302752	62
RXRA	0.05559861	0.446902655	56
MYC	0.0185893	0.452914798	54
EGFR	0.026680775	0.448888889	54
NFKB1	0.015309314	0.434408602	52
RB1	0.020614162	0.443956044	50
CCND1	0.017047903	0.441048035	50
MAPK8	0.014205819	0.441048035	50
CAV1	0.027362368	0.440087146	48
PRKCA	0.025535977	0.436285097	46
AR	0.019027069	0.446902655	44
PPARA	0.022521169	0.438177874	38
PPARG	0.013894734	0.4217119	38
CDK1	0.024538651	0.431623932	36

### Plotting KM curves

**Figure 4:**
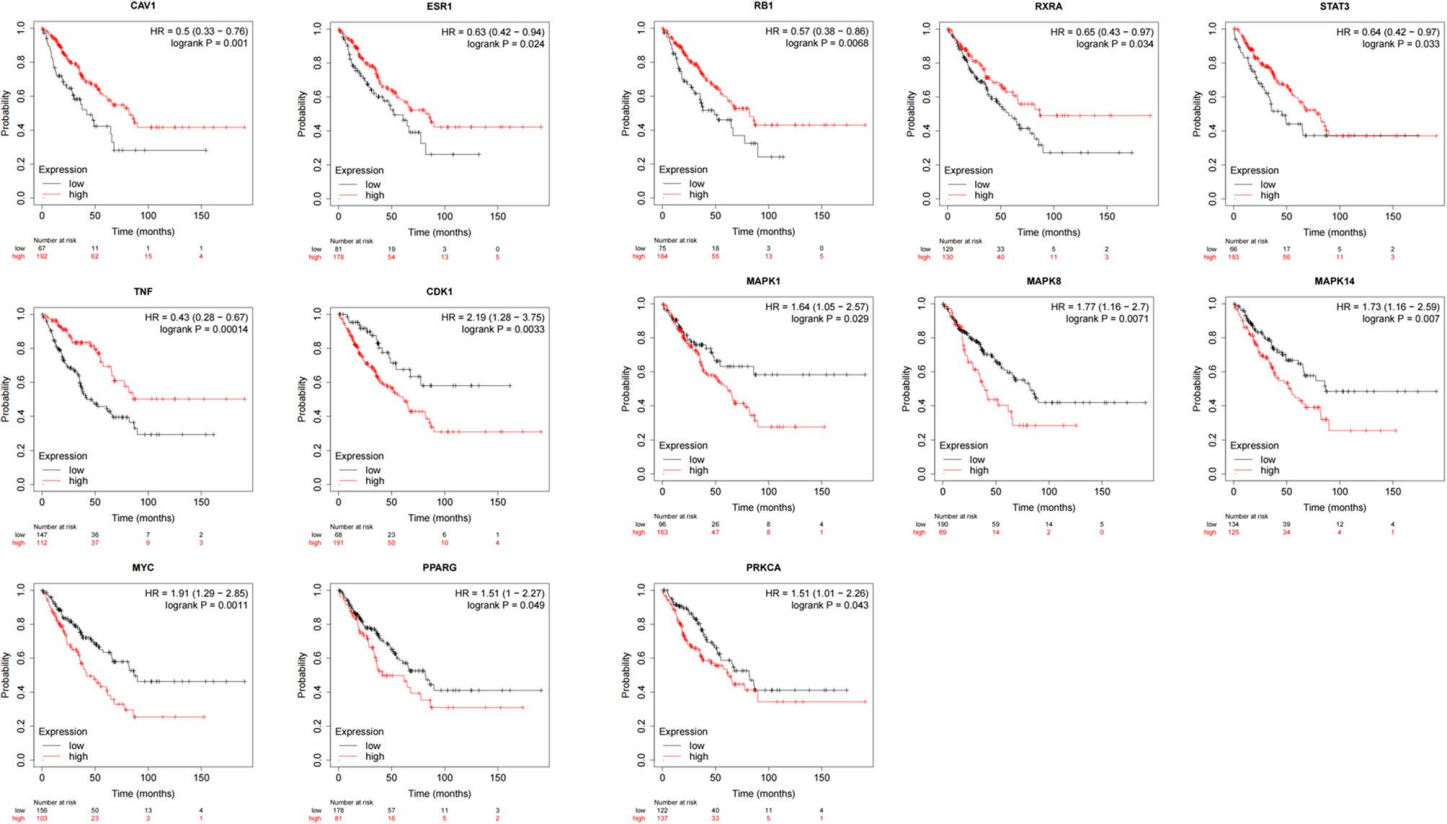
The Kaplan Meier curves. (**A**) (CAV1), (**B**) (ESR1), (**C**) (RB1), (**D**) (RXRA), (**E**) (STAT3), and (**F**) (TNF) are KM curves for genes whose upregulation prolongs median survival in patients with sarcoma. (**G**) (CDK1), (**H**) (MAPK1), (**L**) (MAPK8), (**M**) (MAPK14), (**N**) (MYC), (**O**) (PPARG), and (**P**) (PRKCA) were KM curves for genes whose downregulation could prolong the median survival of osteosarcoma patients.

The hub targets were imported into the Kaplan–Meier Plotter online platform (https://kmplot.com/analysis/), and the KM curves were plotted. The results showed that the differential expression of 13 hub genes had an impact on the overall survival of sarcoma patients (Table 3), and the KM curves of these 13 hub genes are shown in Fig. 4. The targets that improved the overall survival of sarcoma patients when gene expression was upregulated included Caveolin-1 (CAV1), Estrogen receptor (ESR1), Retinoblastoma-associated protein (RB1), RXR-alpha (RXRA), Signal transducer and activator of transcription 3 (STAT3), and Tumor necrosis factor (TNF). The targets that improved overall survival in sarcoma patients when gene expression was downregulated included Cyclin-dependent kinase 1 (CDK1), mitogen-activated protein kinase (MAPK1), Mitogen-activated protein kinase 14 (MAPK14), Mitogen-activated protein kinase 8 (MAPK8), Myc proto-oncogene protein (MYC), Peroxisome proliferator-activated receptor gamma (PPARG), and PRKCA-binding protein (PRKCA).

**Table 3:** Characteristics of the 13 hub genes that had an impact on the overall survival of sarcoma patients.

Name	Betweenness centrality	Closeness centrality	Degree
MAPK1	0.047748112	0.482100239	88
STAT3	0.049144241	0.477541371	88
TNF	0.026999842	0.433476395	66
ESR1	0.026659048	0.464367816	64
MAPK14	0.017013089	0.458049887	64
RXRA	0.05559861	0.446902655	56
MYC	0.0185893	0.452914798	54
RB1	0.020614162	0.443956044	50
MAPK8	0.014205819	0.441048035	50
CAV1	0.027362368	0.440087146	48
PRKCA	0.025535977	0.436285097	46
PPARG	0.013894734	0.4217119	38
CDK1	0.024538651	0.431623932	36

### GO and KEGG enrichment analysis

**Figure 5:**
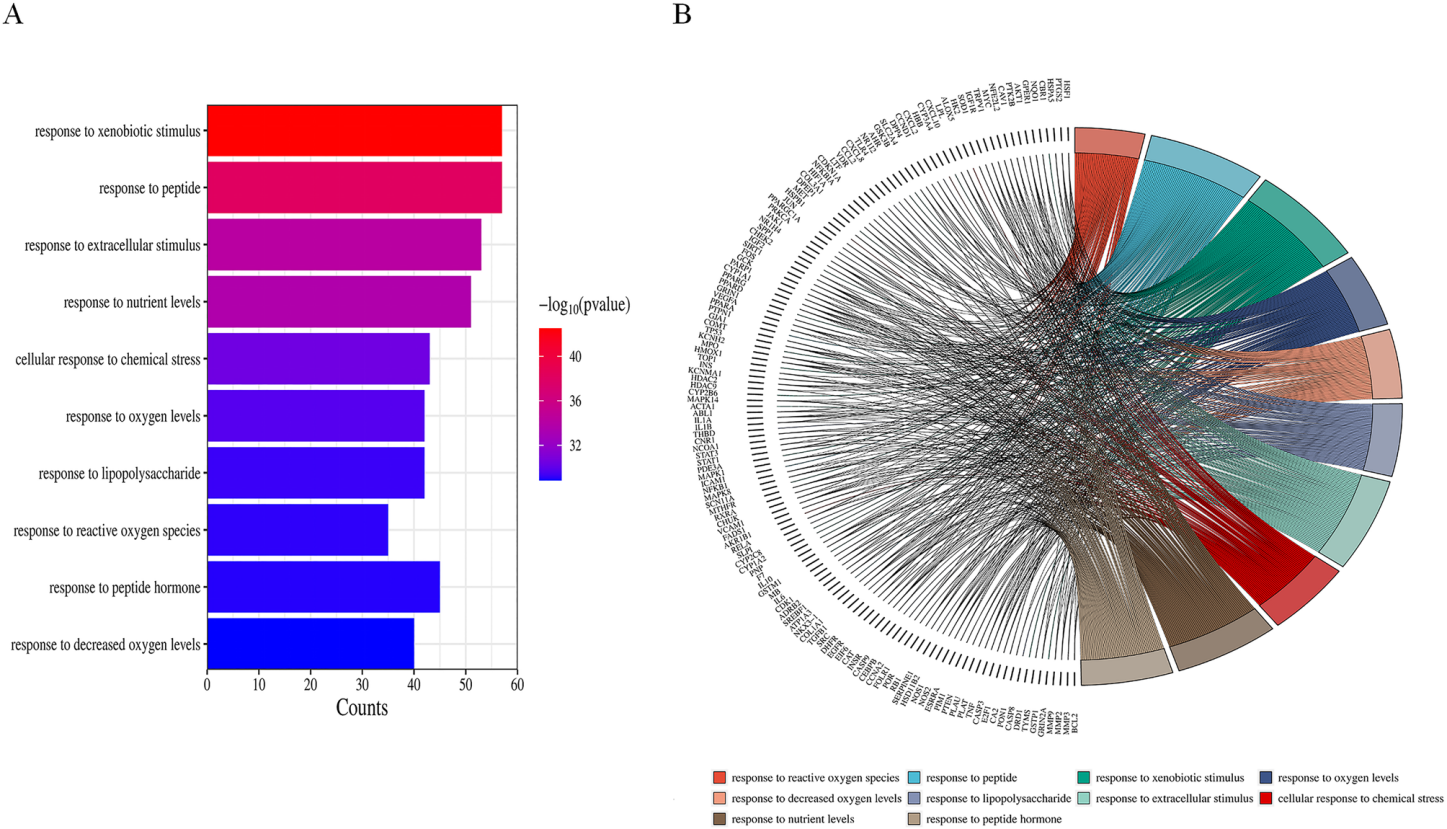
Top ten significant biological process (BP) entries. (**A**) GO enrichment analysis of therapeutic targets for biological process. (**B**): Relationship between the therapeutic targets and biological process.

**Figure 6:**
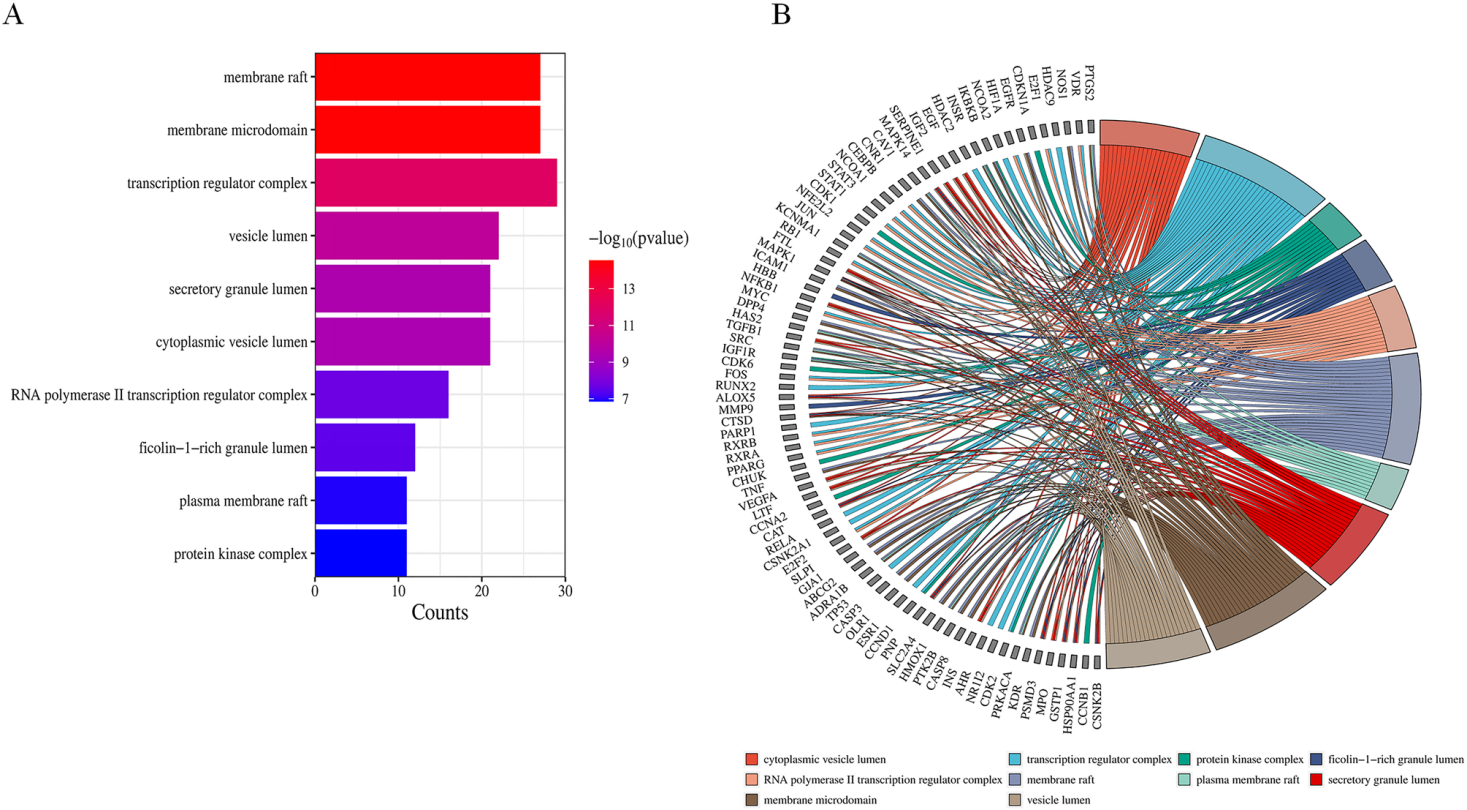
Top ten significant cell component (CC) entries. (**A**) GO enrichment analysis of therapeutic targets for cell component. (**B**) Relationship between the therapeutic targets and cell component.

**Figure 7:**
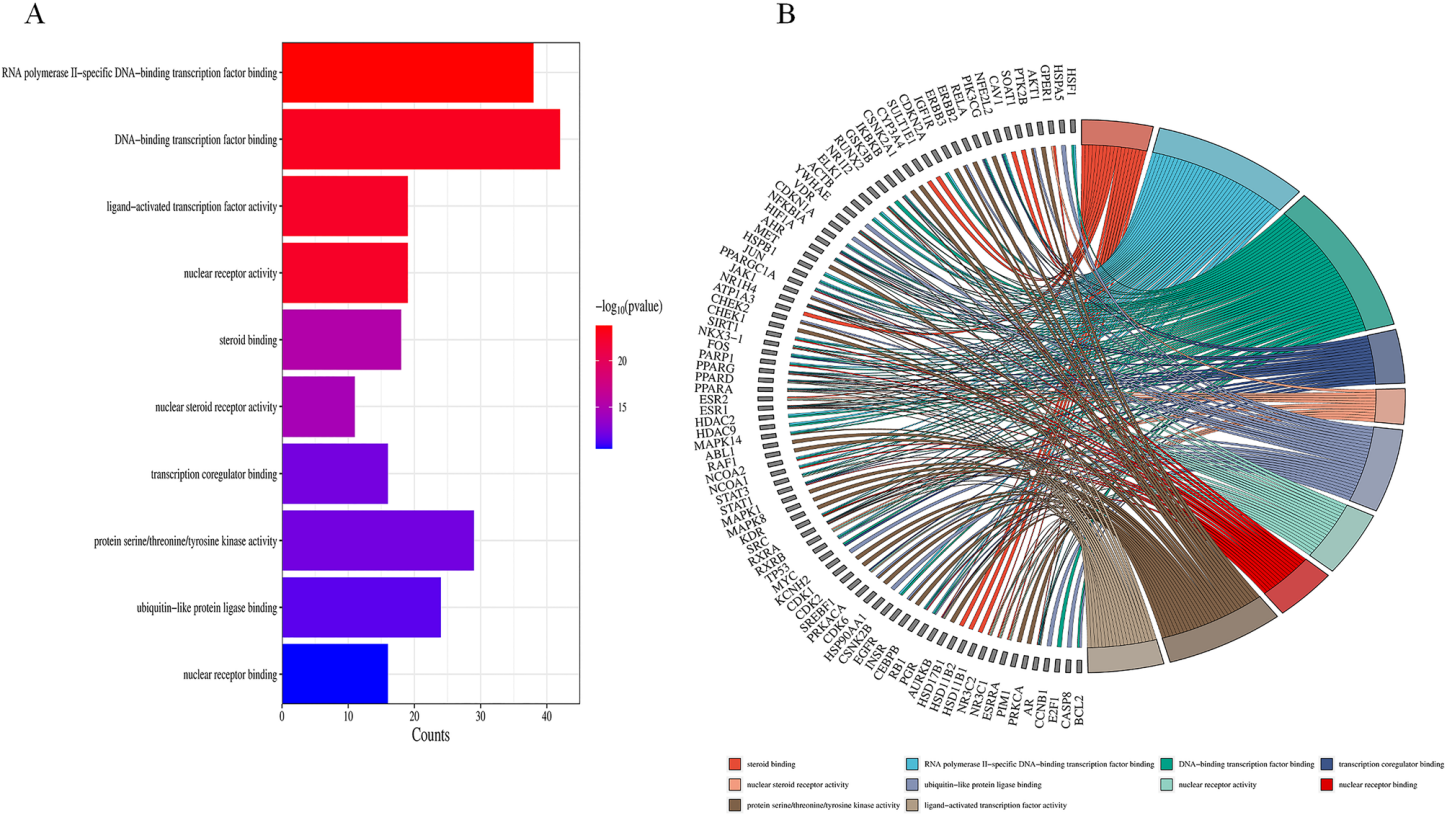
Top ten significant molecular function (MF) entries. (**A**) GO enrichment analysis of therapeutic targets for molecular function. (**B**) Relationship between the therapeutic targets and molecular function.

**Figure 8:**
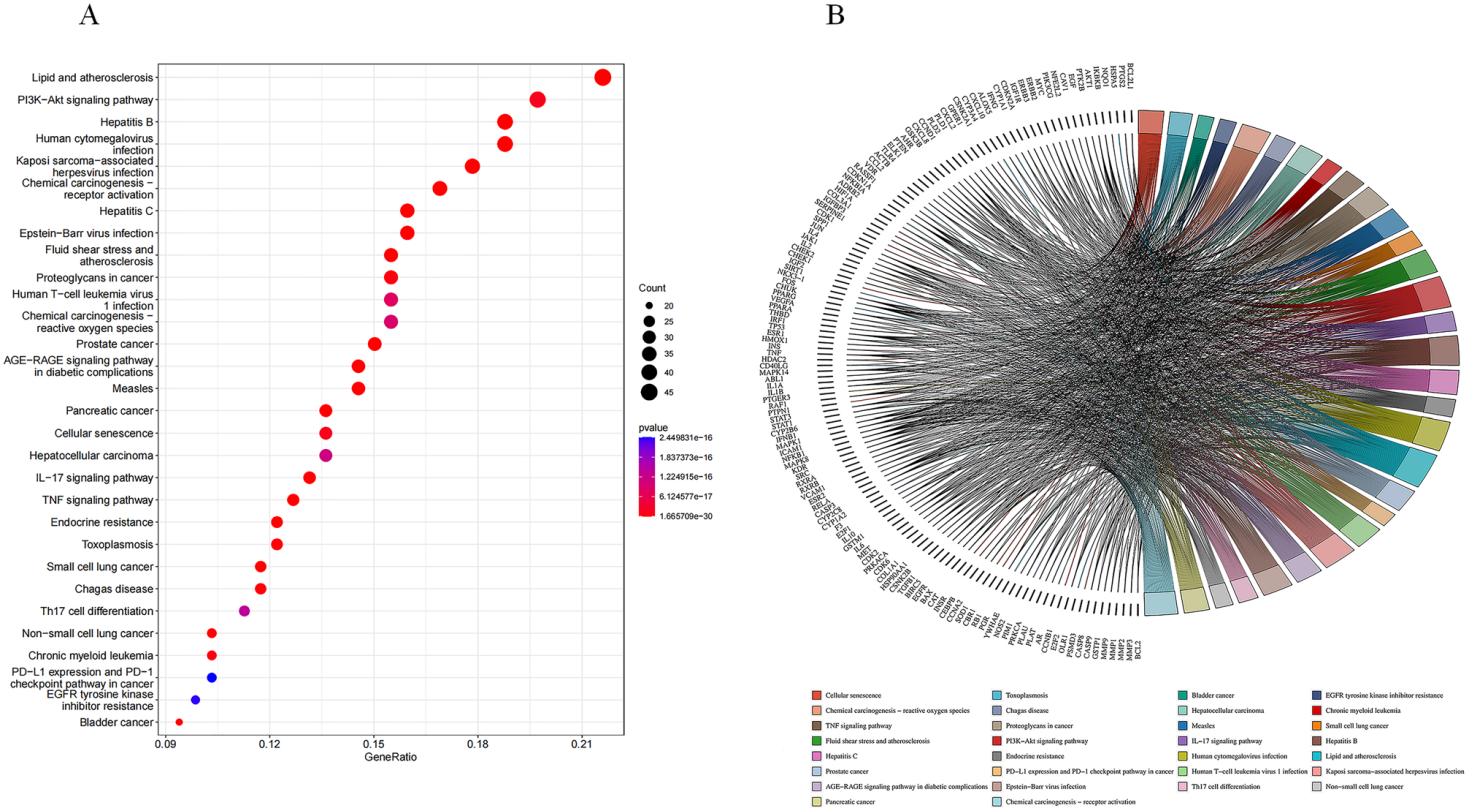
KEGG enrichment analysis for therapeutic targets. (**A**) KEGG enrichment analysis of therapeutic targets for signaling pathway. (**B**) Relationship between the therapeutic targets and signaling pathway.

To investigate the potential functions of Radix Astragali for osteosarcoma, we subjected the potential targets of Radix Astragali for osteosarcoma to GO enrichment analysis, and we presented the results of GO enrichment analysis in terms of BP, CC, and MF. p-values are arranged from smallest to largest, and the top 10 BP, CC, and MF are shown in Fig. 5A,B; Fig. 6A,B and Fig. 7A,B. Figures 5B, 6B, and 7B highlight the genes and relationship between functions. To explore the potential pathways through which Radix Astragali treats osteosarcoma, we subjected the potential targets of Radix Astragali treating osteosarcoma to KEGG enrichment analysis, with the p- values arranged from smallest to largest, and the top 30 results of the enrichment results are displayed in Fig. 8A,B. Figure 8B highlight the genes and relationship between the signaling pathways.

### Molecular docking

**Figure 9:**
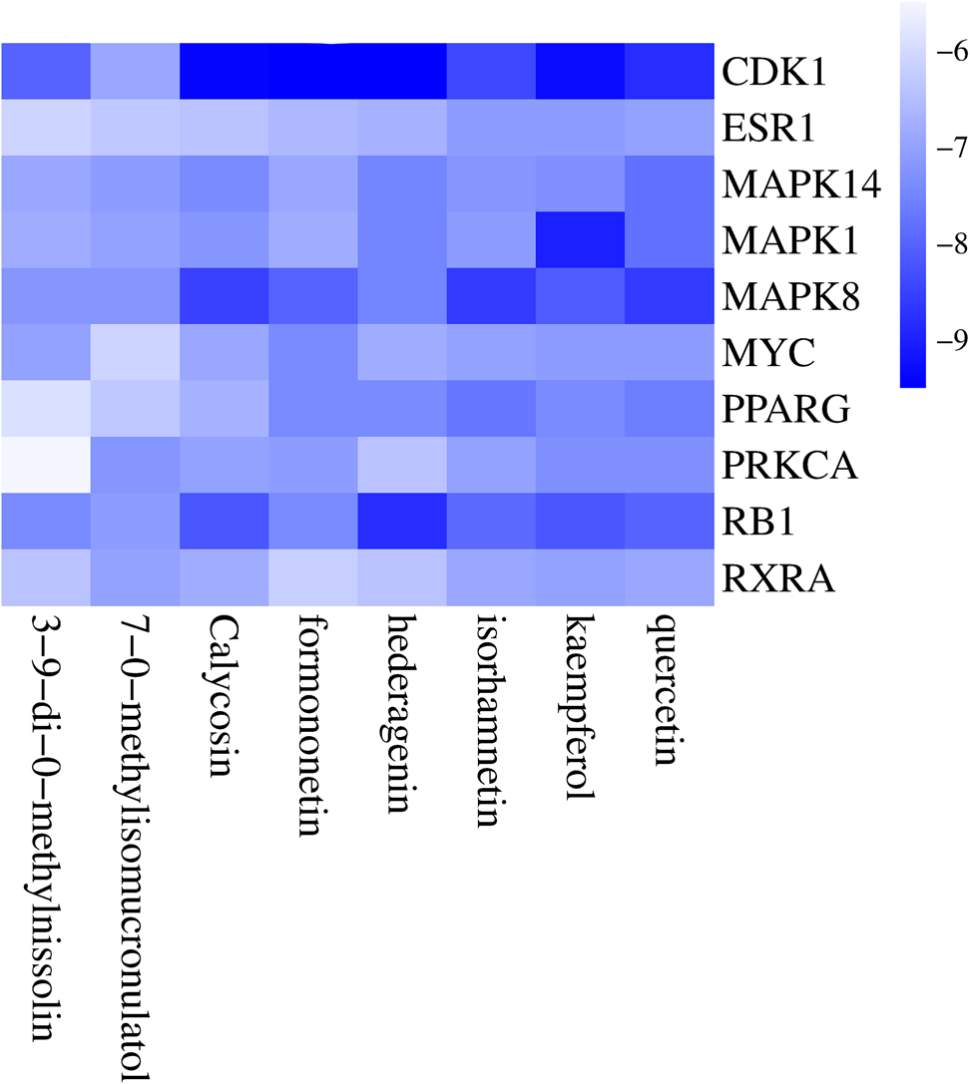
Heatmaps of the docking scores of hub targets combined with corresponding bioactive compound of Radix Astragali. The darker the blue, the more free energy the bioactive ingredient has to bind to the hub targets.

**Figure 10:**
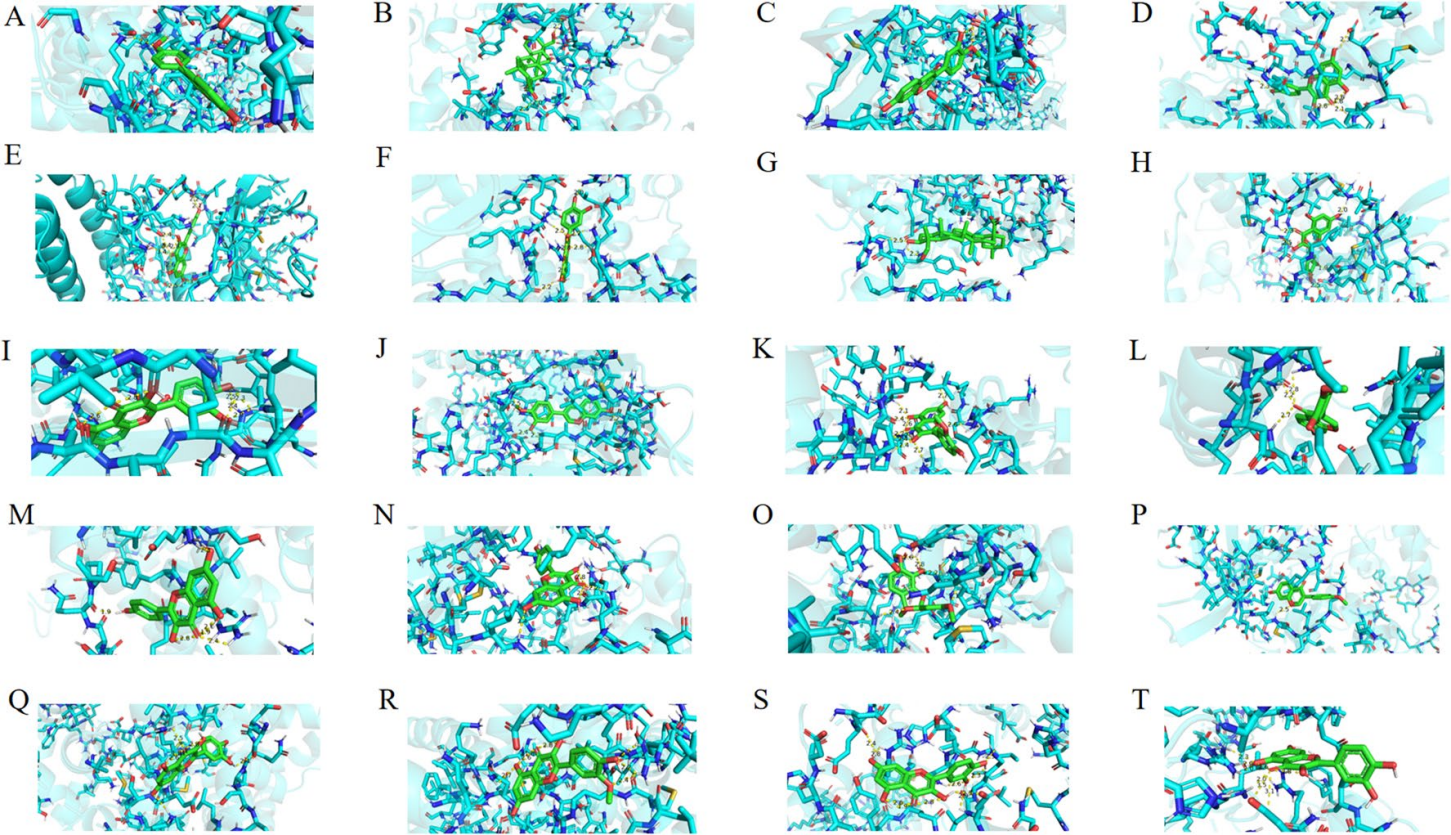
The top twenty significant molecular docking. (**A**) (CDK1, formononetin, − 9.5 kcal/mol); (**B**) (CDK1, hederagenin, − 9.5 kcal/mol); (**C**) (CDK1, Calycosin, − 9.4 kcal/mol); (**D**) (CDK1, kaempferol, − 9.3 kcal/mol); (**E**) (MAPK1, kaempferol, − 9 kcal/mol); (**F**) (CDK1, quercetin, − 8.8 kcal/mol), (**G**) (RB1, hederagenin, − 8.8 kcal/mol); (**H**) (MAPK8, isorhamnetin, − 8.6 kcal/mol), (**I**) (MAPK8, quercetin, − 8.6 kcal/mol); (**J**) (MAPK8, Calycosin, − 8.5 kcal/mol), (**K**) (CDK1, isorhamnetin, − 8.4 kcal/mol); (**L**) (RB1, Calycosin, − 8.2 kcal/mol), (**M**) (RB1, kaempferol, − 8.2 kcal/mol); (**N**) (MAPK8, kaempferol, − 8.1 kcal/mol); (**O**) (CDK1, 3,9-di-O-methylnissolin, − 8 kcal/mol); (**P**) (MAPK8, formononetin, − 8 kcal/mol); (**Q**) (RB1, quercetin, − 8 kcal/mol); (**R**) ((RB1, isorhamnetin, − 7.9 kcal/mol); (**S**) (MAPK14, quercetin, − 7.8 kcal/mol); (**T**) (MAPK1, quercetin, − 7.8 kcal/mol).

To simulate the process of mutual binding between the hub target whose differential expression of the target has an impact on the survival rate of sarcoma patients and the corresponding Astragalus active ingredient, we did molecular docking, and the docking with the free energy of release < − 7 kcal/mol indicated that the corresponding active ingredient and the target and bound effectively in the natural state. Based on the docking results, we plotted the heat map (Fig. 9), and we visualized the docking results for the 20 docks with the most free energy released (Fig. 10), and the basic information of the docking results was displayed in Table 4.

**Table 4:** Information on the docking results of the top 20 significant molecules.

Receptor	Ligands	Free energy (kcal/mol)	Corresponding serial numbers in Fig. 10
CDK1	Formononetin	− 9.5	A
CDK1	Hederagenin	− 9.5	B
CDK1	Calycosin	− 9.4	C
CDK1	Kaempferol	− 9.3	D
MAPK1	Kaempferol	− 9	E
CDK1	Quercetin	− 8.8	F
RB1	Hederagenin	− 8.8	G
MAPK8	Isorhamnetin	− 8.6	H
MAPK8	Quercetin	− 8.6	I
MAPK8	Calycosin	− 8.5	J
CDK1	Isorhamnetin	− 8.4	K
RB1	Calycosin	− 8.2	L
RB1	Kaempferol	− 8.2	M
MAPK8	Kaempferol	− 8.1	N
CDK1	3-9-di-0-methylnissolin	−8	O
MAPK8	Formononetin	− 8	P
RB1	Quercetin	− 8	Q
RB1	Isorhamnetin	− 7.9	R
MAPK14	Quercetin	− 7.8	S
MAPK1	Quercetin	− 7.8	T

## Discussion

Osteosarcoma is one of the common malignant bone tumors in adolescents and children^19^, most often occurring in the epiphysis of long stem bones^20^. The current clinical treatment is so-called "sandwich therapy", which consists of neoadjuvant chemotherapy, radical resection surgery and adjuvant chemotherapy^4,20^. The 5-year overall survival rate of primary OS patients who receive classical treatment is approximately 60–70%^6^, but once distant metastasis occurs, the 5-year survival rate of patients decreases to less than 15%^7^. Radix Astragali is one of the traditional Chinese medicines and its various components have been shown to affect the biological behavior of tumor cells^11,13,14,21–25^, but its effects on osteosarcoma and the mechanisms have not been reported in the literature. By network pharmacology techniques, this study demonstrated that Radix Astragali acts on osteosarcoma through a signaling network formed by hub targets connecting multiple signaling pathways.

In this study, By analyzing the PPI network of potential targets of Radix Astragali for osteosarcoma, we obtained 25 hub targets, including CAV1, ESR1, RB1, RXRA, STAT3, etc. By constructing KM curves, we screened 13 targets that have an impact on the overall survival of sarcoma, including CDK1, MAPK1, MAPK14, MAPK8, MYC, PPARG, PRKCA, etc. The results showed that the 5-year survival rate of sarcoma patients was effectively improved when the expression of genes CAV1, ESR1, RB1, RXRA, STAT3, and TNF was upregulated, and the genes CDK1, MAPK1, MAPK14, MAPK8, MYC, PPARG and PRKCA were downregulated. By analyzing the KEGG enrichment results, we know that Radix Astragali acts on osteosarcoma through multiple signaling pathways, such as IL-17 signaling pathway, PI3K-Akt signaling pathway, TNF signaling pathway and PD-L1 expression and PD-1 checkpoint pathway in cancer. Meanwhile, according to GO enrichment results, GO results suggest that potential therapeutic targets of Radix Astragali for osteosarcoma have multiple molecular functions, such as RNA polymerase II-specific DNA-binding transcription factor binding, DNA-binding transcription factor binding, nuclear receptor activity, and ligand-activated transcription factor activity, etc. Studies have shown that these molecular functions are closely related to the transcriptional processes of tumor cells, cell proliferation, apoptosis, migration, and infiltration^26–29^. According to KEGG enrichment results, multiple hub targets are involved in the signaling process of numerous signaling pathways, such as RELA is involved in the IL-17 signaling pathway, PI3K-Akt signaling pathway, TNF signaling pathway and PD-L1 expression and PD-1 checkpoint pathway in cancer, while TNF is involved in IL-17 signaling pathway and TNF signaling pathway. For example, activation of the PI3K-Akt signaling pathway inhibits proliferation, migration, and infiltration of tumor cells and promotes apoptosis^30–32^, while the TNF signaling pathway inhibits proliferation, migration, and infiltration of tumor cells and promotes apoptosis^33,34^. Thus, it can be concluded that hub targets connect multiple signaling networks to form a signaling network, and Radix Astragali regulates the whole signaling network by acting on the hub targets, and regulates the biological behavior of tumor cells, such as apoptosis, proliferation, migration, and infiltration,etc. To explore the docking of Radix Astragali’s drug ingredients and hub targets, we used the molecular docking technique to simulate the binding process of the hub targets with the corresponding drug ingredients. When the free energy of docking released is < − 7 kcal/mol represents that the ligand and receptor can bind freely in the natural state, we visualized the top 20 docking results with the most free energy released. Among the docking results, quercetin effectively bound to the most hub targets and was the most potential ingredient to become a therapeutic drug acted on osteosarcoma, while among the hub targets, PPARG, RXRA, and ESR1 were able to effectively dock with the most drug ingredients and were the most promising therapeutic targets when Radix Astragali acted on osteosarcoma.

Through pharmacological and molecular docking assays, he regulatory effect of Radix Astragali on osteosarcoma. As we all know, network pharmacology is an efficient tool for the investigation of drug mechanisms. However, it is clear that the present study still has some limitations. Firstly, In vitro and in vivo experiments were not conducted in this study, and the effect of Radix Astragali on osteosarcoma in the In vitro and in vivo situations was not explored, but we have predicted which pathways are targeted by Radix Astragali via network pharmacology, molecular biological validation is our next step. Secondly, each database has a different focus, and there are differences between databases; therefore, there may be potential risks in the joint analysis of multiple databases. Therefore, the discovery of new targets and pathways still needs to be done through basic laboratory experiments. Lastly, Because the molecular docking technique in this study can only simulate the process of free binding between the small molecule and the target when the free energy released from the binding of the two is < − 7 kcal/mol, it indicates that the two can be bound in the natural state, but it cannot predict the effect of the binding of small molecules and the target on the target, so it cannot predict the effect of the small molecule on the target directionality, and the effect of the directionality of the expression of the target still needs to be further explored by basic experiments, we will pursue these experiments in future work.

## Conclusion

We found that Radix Astragali acts on several hub targets that can prolong the survival time of osteosarcoma patients, thus regulating the signaling network formed by several signaling pathways, than achieved the effect of regulating the biological behavior of osteosarcoma cells, such as proliferation, apoptosis, migration, and infiltration, etc. Among the drug ingredients of Radix Astragali, quercetin has the most potential to become an anti-osteosarcoma drug, while the hub targets PPARG, RXRA, and ESR1 are the most potential therapeutic targets when Radix Astragali acting on osteosarcoma.

## Data Availability

The original contributions presented in the study are included in the article Material, further inquiries can be directed to the corresponding authors. TCMSP database: https://old.tcmsp-e.com/tcmsp.php; ETCM database: http://www.tcmip.cn/ETCM/; Symmap database: http://www.symmap.org/; Genecard database: https://www.genecards.org/.

## Abbreviations

BP: Biological processes
CC: Cellular components
CDK1: Cyclin-dependent kinase 1
DL: Drug likeness
ESR1: Estrogen receptor
GO: Gene ontology
KEGG: Kyoto encyclopedia of genes and genomes
KM: Kaplan–Meier
MAPK14: Mitogen-activated protein kinase 14
MF: Molecular functions
MAPK1: Mitogen-activated protein kinase
OB: Oral bioavailability
PPARG: Peroxisome proliferator-activated receptor gamma
PPI: Protein–protein interaction
RB1: Retinoblastoma-associated protein
RXRA: RXR-alpha
TNF: Tumor necrosis factor
CAV1: Caveolin-1
STAT3: Signal transducer and activator of transcription 3
MAPK8: Mitogen-activated protein kinase 8
MYC: Myc proto-oncogene protein
PRKCA: PRKCA-binding protein
PPI: Protein–Protein Interaction
TCMSP: Traditional Chinese medicine systems pharmacology database and analysis platform
ETCM: The encyclopedia of traditional chinese medicine
TCM: Traditional chinese medicine
PDB: The protein data bank
3D: Three-dimensional
2D: Two-dimensional
